# Role or Synergistic Interaction of Adenosine and Vitamin D3 Alongside High-Intensity Interval Training and Isocaloric Moderate Intensity Training on Metabolic Parameters: Protocol for an Experimental Study

**DOI:** 10.2196/10753

**Published:** 2019-01-30

**Authors:** Seyed Javad Mirghani, Maghsoud Peeri, Omid Yaghoobpour Yekani, Masoud Zamani, Foad Feizolahi, Sina Nikbin, Armin Derakhshideh, Niloufar Mousavi, Zohreh Khojasteh, Zeynab Nasrollahi, Elya Khorasani, Elham Ghodousi Johari, Tayebeh Afshar, Mohammad Ali Azarbayjani

**Affiliations:** 1 Department of Exercise Physiology Faculty of Physical Education and Sports Science Islamic Azad University, Central Tehran Branch Tehran Islamic Republic of Iran; 2 Department of Exercise Physiology Islamic Azad University, Science and Research Branch Tehran Islamic Republic of Iran; 3 Department of Physical Education and Sport Science Islamic Azad University, Karaj Branch Karaj Islamic Republic of Iran

**Keywords:** high-fat, diet-induced obesity, high-intensity interval training, isocaloric moderate-intensity training, vitamin D3, adenosine, metabolic parameters, weight loss

## Abstract

**Background:**

Obesity is known as one of the major causes of epidemiologic diseases worldwide; therefore, the introduction of treatment strategies by medical professionals, such as the use of various medicines and exercise programs to reduce fat or prevent obesity, is on the rise. Recently, researchers have shown special interest in assessing the effect of lipolytic adenosine and vitamin D deficiency, as well as the effect of exercise, on decreasing body fat percentage.

**Objective:**

This study has been designed to examine the effect of adenosine and vitamin D3 injections, in conjunction with high-intensity interval training and isocaloric moderate-intensity training, on the metabolic parameters of obesity induced by a high-fat diet.

**Methods:**

This is an experimental study using 92 Wistar rats. At 6 weeks of age, the rats' weights will be recorded, after which they will have 1 week to adapt to their new environment before being divided into 12 groups. The rats will participate in a 2-stage experimental intervention, including a 13-week fattening diet phase followed by a 12-week exercise training phase consisting of an exercise program and the injection of adenosine and vitamin D3. Groups 1 and 2 will have a normal diet, and the other groups will have a diet of 40% fat, with free access to food and water up to the second half of the second stage of the study (end of the sixth week of training). After termination of the interventions, tissue collection and molecular assessments (blood for biochemical, tissues for gene expression analyses, and anthropometrical indexes) will be performed.

**Results:**

The project was initiated in April 2017 and completed in December 2017. Data analysis is under way, and the first results are expected to be submitted for publication in November 2018.

**Conclusions:**

We hypothesize that weight loss–induced molecular changes and upregulation will be observed in line with an increase in lipolysis and beta oxidation in muscle and fat tissue as a result of performing isocaloric training in drug-receiving rats and groups on a high-fat diet.

**International Registered Report Identifier (IRRID):**

RR1-10.2196/10753

## Introduction

### Background

Obesity is a main health risk factor [1,2] and the major cause of diseases, including metabolic syndrome [3], type 2 diabetes, high blood pressure, and cardiovascular incidence worldwide. The high intake of energy within the body results in abnormal accumulation of fat in adipose tissues [4], which has deteriorating effects on health, life quality, and aging [5]. Thus, the damage of glucose and fat metabolism pathways and the disturbance in metabolic balance of these interrupted conditions [6-8] result in the incidence or development of fat-related diseases [9].

Plant-based substances [4,10,11] and medicines [12-16] have been proposed as strategic treatment and preventive measures for obesity. Presently, one of the most challenging issues in the field of pharmacy is discovering the most effective antiobesity intervention with the least negative side effects on humans. Recently, researchers have focused on the effect of the most active forms of vitamin D and have shown fatty acid oxidation [17] and its controlling role in the incidence of obesity [13,18-23]. On the other hand, while some studies have shown that adipogenesis [24,25] continues through different mechanisms, others have focused on identifying intervention factors that can lead to weight loss, particularly, fat weight, with the researchers revealing the undetected effects of adenosine molecules driven from adenosine triphosphate (ATP) [26].

Depending on the type and dependency of adenosine on specific G proteins, it has a link with one of the 4 receptors—A1, A2A, A2B, and A3—in different tissues showing different functions [27-31]. In contrast to the clinical findings, exercise science training experts rely on the effective and preventive effects of different types of exercise programs on obesity that are relatively consistent without any harmful side effects. On the other hand, considering the significance of intensity and duration of exercise training programs [32-41], high-intensity interval training (HIIT) programs have been identified as a fat controlling intervention [33]. The control of weight increase due to the high-fat content of diet [34] compared with stable aerobic activity, improvement in fat distribution, and insulin with similar energy cost [35], has an effect on obesity. Regardless of the benefits of physical activity on the improvement of obesity, there are inconsistent findings with regard to the decrease in fat indices through participation in physical activity without calorie restriction. In addition, there are a limited number of studies on the significance of calorie consumption based on exercise training volume in contrast to response to different types of training, leading to the same changes in metabolic conditions of obesity-induced high-fat diet with participation in HIIT and isocaloric moderate-intensity training as observed earlier [36].

Thus, considering the significance of finding an antiobesity medicine to reduce weight with less harmful side effects and the undeniable effect of exercise as medicine [14,37] for health and longevity, it seems necessary to examine and compare the effect of medicine, exercise, and their interaction on health. Therefore, due to the inadequate knowledge of introducing harmless medicine to control the increase of the volume and size of fat cells and enhance fat-burning activities in high-fat diets, it seems adenosine (by activating adenosine receptors in response to the density and release of adenosine within the cell that leads to different processes of fat burning) and vitamin D injections, in conjunction with isocaloric sport training, may play a significant role in the reduction of fat accumulation, lipolysis regulation, and insulin sensitivity in vital metabolic organs including the liver, muscles, and different fat tissues, which may eventually lead to weight loss.

### Study Objective

The aim of this study is to examine the interaction of adenosine and vitamin D3 alongside HIIT and isocaloric moderate-intensity training on anthropometric, thermogenic, and metabolic gene parameters in high-fat diet–induced obese rats.

## Methods

### Animals

In this experimental protocol, 92 male Wistar rats will be prepared by the Shahid Mirghani Research Institute. This study has been reviewed by the research ethics committee of Sports Science Research Institute and was approved with the code IR.SSRI.REC.1395.115. The rats will be kept in similar laboratory environment conditions at 22°C±3°C in a 12-hour day-night cycle. All rats will be fed a normal diet until 5 to 6 weeks to gain 182.32 grams of weight. After 1 week of adaptation to a new environment, the 92 rats will be divided into 12 groups to participate in the 2 stages of the experimental intervention, including a 13-week fattening diet plan (they will consume 40% fat) followed by a 12-week exercise program. All the rats in the normal diet (except groups 1 and 2) will have free access to food and water up to the second half of the second stage (end of the sixth week of training). In the beginning of the seventh week, the amount of food given to all the groups will be prepared based on a gram scale (based on the mean value of food in groups 3 and 6) for 6 weeks in an identical scale. This process will continue until the end of the training stage. Anthropometric measures will be assessed for all groups, including weight per week, body mass index (BMI), waist and chest size and ratio, height, Lee index, calories consumed, the ratio of weight gain to the total amount of food consumed, the ratio of weight gain to the total calories consumed monthly, and the amount of food consumed daily by every rat.

### Diet

Normal diet will contain 4.30 kcal per gram including 3.87% fat (soy oil), 17.46% casein protein, 68.7% carbohydrates, 8.97% minerals, and 1% vitamins. High-fat diet will contain 5.81 kcal per gram with 40% fat (20% soy oil and 20% [animal fat] subcutaneous fat oil), 14.1% casein protein, 36.58% carbohydrates, 8.4% minerals, and 0.72% vitamins.

### Experimental Groups

**Figure 1:**
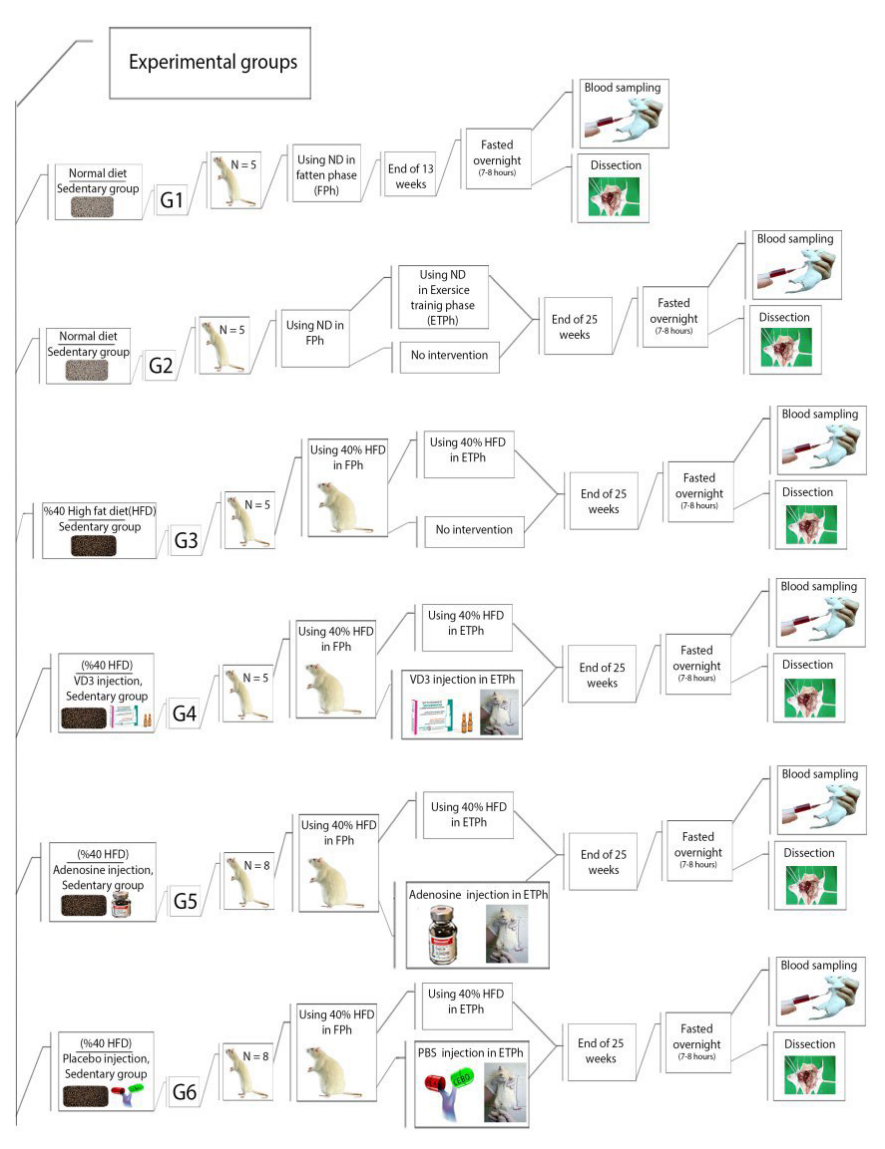
Experimental groups’ timeline and procedures: Part 1. Group 1 is the control group and will be slaughter after the first stage (n=5); Group 2 is the second control group (n=5), which will still be fed a normal diet; Group 3 will continue on a high-fat diet (n=5); Group 4 will continue on a high-fat diet and vitamin D3 injection (n=5); Group 5 will continue on a high-fat diet and adenosine injection (n=8); Group 6 will continue on a high-fat diet and placebo injection (n=8). ND: normal diet, FPh: fatten phase, ETPh: exercise training phase, HFD: high-fat diet.

**Figure 2:**
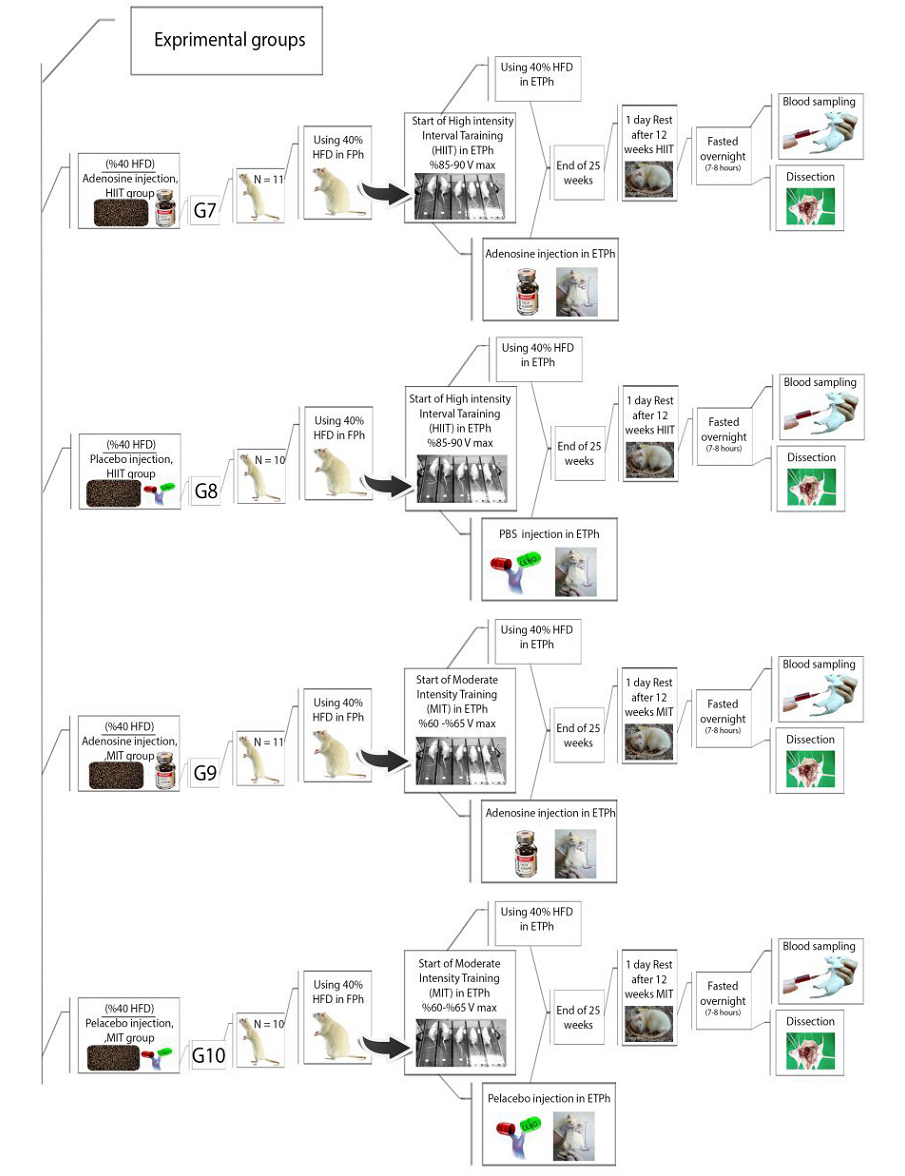
Experimental groups’ timeline and procedures: Part 2. Group 7 will continue on a high-fat diet and undergo HIIT (n=11); Group 8 will continue on a high-fat diet and undergo HIIT and placebo injection (n=10); Group 9 will continue on a high-fat diet and undergo moderate-interval training (MIT) and adenosine injection (n=11); Group 10 will continue on a high-fat diet and undergo MIT and placebo injection (n=10). HFD: high-fat diet, FPh: fatten phase, HIIT: high intensity-interval training, ETPh: exercise training phase, MIT: moderate-intensity training.

**Figure 3:**
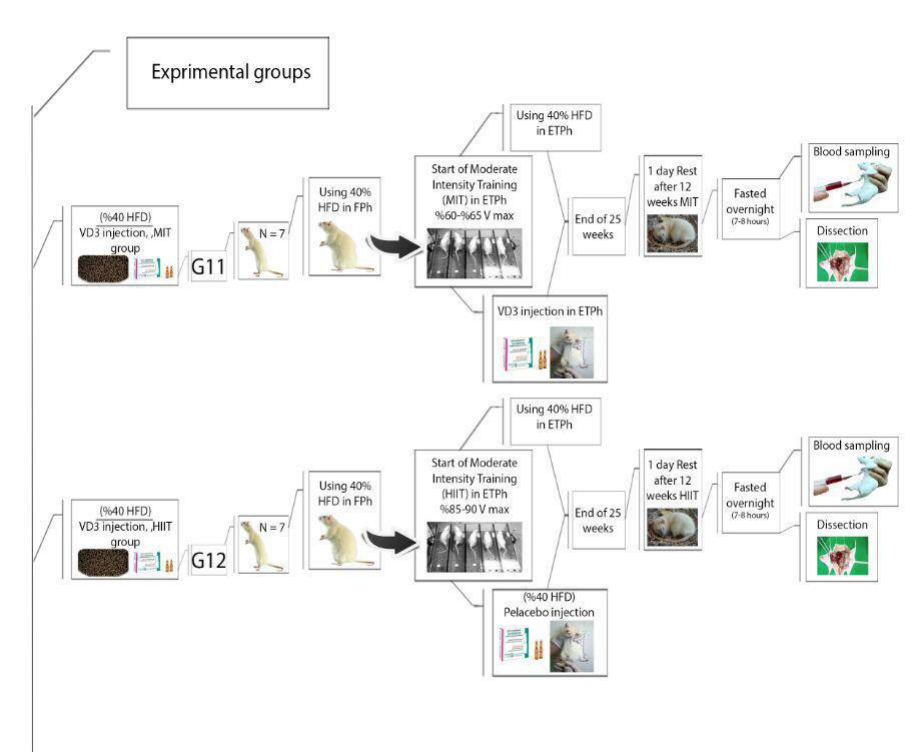
Experimental groups’ timeline and procedures: Part 3. Group 11 will continue on a high-fat diet and undergo MIT with D3 injection (n=7); Group 12 will continue on a high-fat diet and undergo HIIT with D3 injection. HFD: high-fat diet, ETPh: exercise training phase, MIT: moderate-intensity training, HIIT: high intensity-interval training.

**Figure 4:**
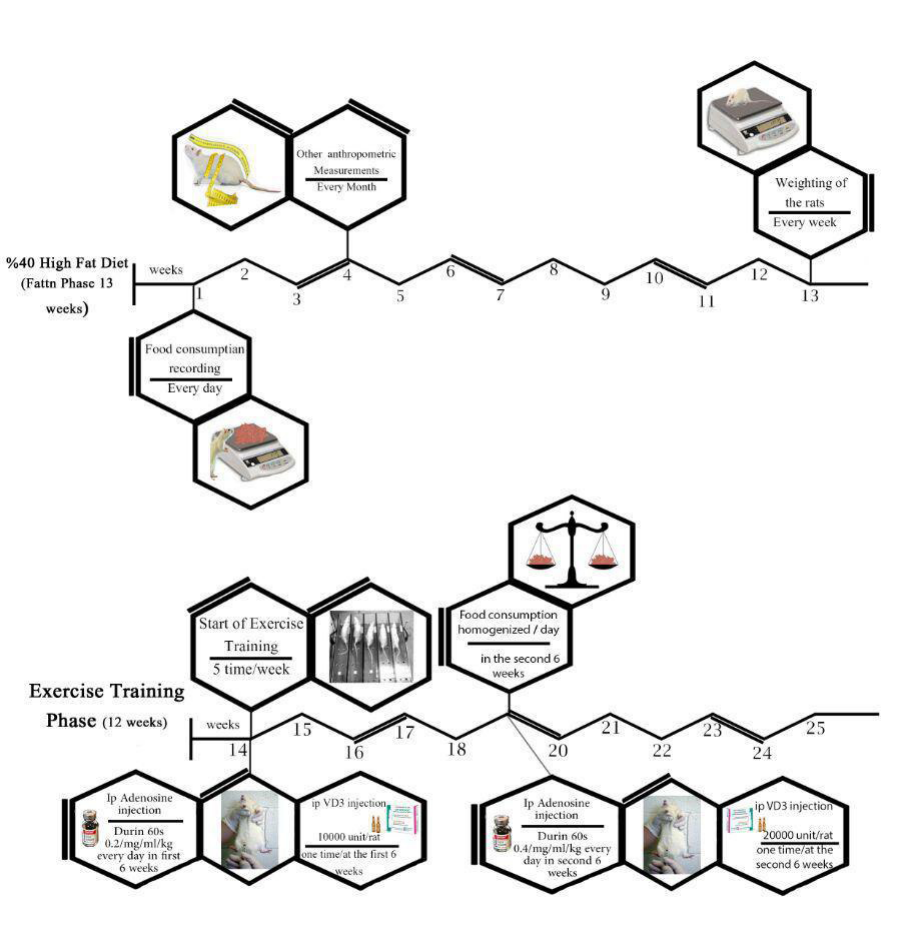
Experimental outline: Fatten Phase. IP: intraperitoneal.

Before any intervention, all 92 rats will be assigned randomly to 12 groups while matched for their weights. Moreover, 2 of these groups will serve as the control group and receive a normal diet. The remaining groups will go through 2 treatment stages; in the first stage, they will consume a 40% fat content diet for 13 weeks. In the second stage of the protocol (training phase), 1 of the control groups (n=5), Group 1, will be slaughtered. The 11 remaining groups will include Group 2, the second control group (n=5), which will still be fed a normal diet; Group 3 will continue on a high-fat diet (n=5); Group 4 will continue on a high-fat diet and vitamin D3 injection (n=5); Group 5 will continue on a high-fat diet and adenosine injection (n=8); Group 6 will continue on a high-fat diet and placebo injection (n=8); Group 7 will continue on a high-fat diet and undergo HIIT (n=11); Group 8 will continue on a high-fat diet and undergo HIIT and placebo injection (n=10); Group 9 will continue on a high-fat diet and undergo moderate-interval training (MIT) and adenosine injection (n=11); Group 10 will continue on a high-fat diet and undergo MIT and placebo injection (n=10); Group 11 will continue on a high-fat diet and undergo MIT with D3 injection (n=7); and Group 12 will continue on a high-fat diet and undergo HIIT with D3 injection (n=7; Figures 1-4).

### Anthropometric Assessments

The abdominal circumference (immediate anterior to the forefoot), thoracic circumference (immediate behind the foreleg), and body length (nose-to-anus or nose-anus length) will be determined in all the rats every month. The measurements will be done on anaesthetized rats (0.1 mL intraperitoneally of 1% sodium barbiturate). The body weight and body length will be determined with the following anthropometrical parameters [42]:

BMI = body weight in g/length^2^ (cm^2^)

Lee index = cube root of body weight in grams/nose-to-anus length (cm)

Specific rate of body mass gain will be determined by: g/kg = dM/Mdt, where dM represents the gain of body weight during dt = t2−t1, and M is the rat body weight at t1.

### Body Mass and Food Intake

Body mass and food intake (difference between the feed offered and the remaining feed) of each animal will be measured daily throughout the experimental period by precision balance (Gehaka, model BG 2000, Brazil). Feed efficiency and energy efficiency will be calculated using the following formulas:

Feed efficiency = Body mass gain (g)/Total food intake (g)

Energy efficiency = Body mass gain (g)/Total caloric intake (kcal)

### Drug Treatment

A total of 190 doses of 3mg/mL adenosine packs and vitamin D3 with 300,000 IU/mL will be purchased from the College of Pharmacy, Tehran University of Medical Sciences. The dosage of adenosine drugs was calculated based on weight and will be prepared in 1 cc of saline. During the first 6 weeks of the training phase, every rat will be injected intraperitoneal (IP), 0.2 mg/mL/kg adenosine dose and vitamin D3 with 10,000-unit dose per rat. After 6 weeks of training, to assess the rate of the effectiveness of the drug, a crossover design will be employed by introducing a dose of 0.4/mg/mL/kg adenosine injection per day and increased unit dose of vitamin D3 to 20,000 IU/ml once in the beginning of the second 6-weeks training period. These doses will undergo no further changes up to the end of the protocol.

### Adenosine Intraperitoneal Injection Aspects

The adverse effects of adenosine, however, limit the usefulness of this agent as a systemically (intravenously or intra-arterially) administered drug. When so administered, adenosine can cause heart block, asystole, arrhythmias, bradycardia, hypotension, bronchoconstriction, and a stress reaction consisting of flushing, headache, dyspnea, chest pressure, and nausea. This is done via single application or intermittent or continuous peritoneal lavage, which induces beneficial effects on the intestines of a subject. This approach can achieve pharmacologically active levels of adenosine in the intestinal wall of a mammal (including humans) without producing significant levels of adenosine in the systemic circulation of the subject.

However, there is a possibility that the metabolic barrier to adenosine absorption by the gastrointestinal tract, that is, intestinal adenosine deaminase [43] would be so effective in limiting the bioavailability of peritoneally administered adenosine that active levels of adenosine in the gastrointestinal tract could only be achieved with concentrations of adenosine in the peritoneal cavity so high that absorption at other sites in the peritoneal cavity would result in overwhelming systemic levels of adenosine. When administered to a subject by peritoneal lavage, adenosine dilates the splanchnic circulation and increases adenosine levels in the mesenteric vein, without affecting systemic hemodynamics or increasing adenosine levels in the arterial circulation. This invention, therefore, establishes that therapeutically effective levels of adenosine can be achieved in the peritoneal cavity in a subject without attaining pharmacologically active levels in the subject’s systemic circulation [44]. Thus, according to the side effects of intravenous injection, the authors will use IP injection in this study. Moreover, considering the high lethal dose (LD50) of the adenosine and its half-life of 0.6 to 10 seconds and also prolonged absorption through IP injection, we will select 0.2 and 0.4 mg/mL/kg.

### Exercise Protocol

The rats in the training groups will be placed on an animal treadmill to run at various speeds of 6, 8, and 10 m per min in a trial period of a week before the main exercise protocol to become acquainted with the procedures. Then, every rat will be placed on the treadmill to continue running at maximum speed up to the exhaustion point. Following the recording of the maximum speed during exhaustion for every rat [38], the mean value of speed of the exercising rats will be calculated. Then, the exercise protocol will be designed [39] (Tables 1 and 2). The designed program will be based on the data obtained through the pilot phase. The HIIT will include an 85% to 90% V_max_ intensity, whereas, the MIT will be set at 60% to 65% V_max_ level. The warm-up period included comprises 3 min of running at a speed of 10 m per min and cool-down period of 2 min of running at a speed of 15 m per min. Both exercise protocols will be matched for the training volume (consumed calories) to determine the effect of types of exercise programs (isocaloric exercise).

### Outcomes

After selecting the rats based on inclusion criteria (weight, age, and species of rats), we will record anthropometrical parameters each week to monitor the changes and use of the dose of drugs and exercise variables. Finally, metabolic, thermogenic, and lipogenesis genes in metabolic tissue (adipose tissues, muscles, liver, and heart, respectively) will be measured and compared to understand the possibility of changes resulting from each intervention in each group that can lead to weight loss, especially fat.

**Table 1:** High-intensity interval training (HIIT) protocols; 12 weeks high-intensity interval training and 12 weeks isocaloric moderate training; HIIT will be performed 5 times a week with 1 min active/rest ratio.

Week	Bouts, n	Load, meters/minute	Time, minutes	Active rest, minute	Bouts, n	Load, meters/minute	Distance, meters/minute
1	7	31	1	1	6	15	402
2	8	31	1	1	7	15	448
3	8	35	1	1	7	17	494
4	9	36	1	1	8	17	555
5	9	41	1	1	8	19	616
6	9	45	1	1	8	20	660
7	10	45	1	1	9	22	743
8	10	47	1	1	9	22	763
9	10	49	1	1	9	23	792
10	10	50	1	1	9	24	811
11	10	52	1	1	9	24	831
12	10	55	1	1	9	25	870

**Table 2:** Isocaloric moderate-intensity training (MIT); 12 weeks high-intensity interval training (HIIT) and 12 weeks isocaloric moderate training; MIT will be performed 5 times a week with same distance of HIIT as an isocaloric exercise training.

Week	Bouts, n	Load, meters/minute	Time, minute	Distance, meters/minute
1	1	20	15:21	402
2	1	20	17:39	448
3	1	21	19	494
4	1	21	21:54	555
5	1	22	23:41	616
6	1	23	24:34	660
7	1	24	27	743
8	1	24	27:50	763
9	1	24	29:3	792
10	1	24	29:50	811
11	1	24	30:38	831
12	1	25	31	870

### Tissue Collection

After 24 hours of rest and 8 hours of fasting, the rats will be anesthetized by applying pentobarbital sodium (40 mg/kg; IP). Then, after reaching a complete anesthetics condition, blood samples will be drawn directly from the heart and transferred into tubes for serum separation by centrifugation. The samples will be frozen up to −80°C for fat and fat-burning marker analysis. The white fat samples (kidney circumference and visceral), mesenteric (visceral), thigh fat (subcutaneous), interscapular (brown fat), epicardial fat, liver (from the inferior right lobe), gastrocnemius and plantaris muscles, heart epics, and superior part of the thigh will be isolated in 2×2 mm size. All sample collection will be performed from 2 to 4 pm after 7 to 8 hours of fasting. After placing the samples in nitrogen for RNA extraction and gene analysis, they will be transferred to a temperature of −80°C.

### Quantitative Polymerase Chain Reaction

Adipose tissue samples will be homogenized in TRIzol solution using a tissue homogenizer (Tissue-Lyser LT; Qiagen, Valencia, CA). Total RNA will be assayed using a NanoDrop spectrophotometer (Thermo Scientific, Wilmington, DE) to assess purity and concentration. First-strand complementary DNA (cDNA) will be synthesized from total RNA using the high-capacity cDNA reverse transcription kit (Applied system; Applied Biosystems). Primer sequences (available upon request) will be designed using the National Center for Biotechnology Information primer design tool. All primers will be purchased from Pishgam (Pishgam, Iran). A 20 µL reaction mixture containing 10 µL SYBR Green Mastermix (Amplicon) and appropriate concentrations of gene-specific primers plus 1000 ng/µL of cDNA template will be loaded in each well of a 96-well plate. All polymerase chain reactions (PCRs) will be performed in duplicates. PCR will be performed with thermal conditions as follows: 95°C for 10 min, followed by 40 cycles of 95°C for 15 seconds, and 60°C for 45 seconds. A dissociation melt curve analysis will be performed to verify the specificity of the PCR products. Glyceraldehyde-3-phosphate dehydrogenase (GAPDH) primers will be used to amplify the endogenous control product. Messenger RNA expression values will be presented as 2_ΔΔCT. Data will be expressed as the fold difference relative to GAPDH. Candidate variables include lipolytic, lipolysis, and thermogenesis genes in the visceral, subcutaneous, and brown adipose tissue (BAT), gastrocnemius and soleus muscles, heart muscle, and liver tissues.

For examination of the gene expression in every group, real-time PCR will be employed by ABI Applied Biosystems, Real-Time PCR Systems, (StepOne, Hettich Centrifuges, UNIVERSAL 320, Capacity: 4×100 mL | 32×15 mL), Relative Centrifugal Force/revolutions per minute (RPM/RCF): 15,000/21,382, Temperature Control: −20 to +40°C, cDNA Synthesis Kits— Thermo Scientific, Revert Aid First Strand cDNA Synthesis Kit.

### Western Blot Analysis

Radioimmunoprecipitation assay buffer cell lysates will be used to produce western blot-ready samples. Samples will be separated by sodium dodecyl sulfate-polyacrylamide gel analysis transferred to polyvinylidene difluoride membranes, and will be incubated with primary antibodies. Horseradish peroxidase-conjugated mouse or rabbit secondary antibody will be used to detect primary antibodies and will be stained with 3,3’-diaminobenzidine (Sigma-Aldrich, USA). Protein loading will be measured by Bradford (Sigma) staining to determine total protein concentration. The total protein will be loaded in each lane and quantified. These values will be used to adjust for any difference in protein loading or transfer of all band densities. Individual protein bands will be quantified using image J software (National Institutes of Health, USA), and data will be expressed relative to rabbit polyclonal beta actin antibody. Antibodies will be purchased from Abcam (Abcam, Germany).

### Biochemical Analysis

The concentrations of glucose, total triglyceride (TG), total cholesterol, high-density lipoprotein cholesterol (HDL-C), low-density lipoprotein cholesterol, and very low-density lipoprotein cholesterol in serum will be determined by the clinical pathology laboratory using an automated analyzer (Alpha Classic–tajhizatsanjesh). Glycerol, insulin, and free fatty acids will be measured respectively with rat-specific enzyme-linked immunosorbent assay kits (cat No: ZB-GCL48A. Lot.No: ZB-OC717210, cat No: 10-1250-01; Lot No. 25692 ZB-A1515818) according to the manufacturer’s instructions. Quantitative insulin sensitivity check index (QUICKI) and homeostatic model assessment for insulin resistance will be calculated as described previously using the equation: QUICKI51/(log [I0] 1 log [G0]), where I0 is fasting insulin (lU/mL) and G0 is fasting glucose (mg/dL)2.

### Statistical Analysis

The sample size for this research protocol will be estimated based on the effect size that was effective in previous research, and G*power software will be used to determine the required number. For the descriptive results, mean and SD will be calculated and reported in appropriate tables. For any variable showing nonsymmetry or lack of normality, median, 25, and 75 percentiles will be calculated. For determination of the interaction effect, the mean differences and CIs will be calculated, and for estimation of the effect size, Cohen method will be employed to calculate the standardized mean differences. Each intervention will be evaluated by 2-way analysis of variance.

## Results

The project was founded in April 2017 and data collection is expected to be conducted until December 2017. Data analysis will start once the data collection is completed, and the first results are expected to be submitted for publication in November 2018.

## Discussion

### Summary

The rise in obesity has contributed to increasing numbers of people who need to and attempt to lose weight [45]. Thus, most studies have focused on strategies such as caloric restriction [46], intensity of exercise training, and diet combined with exercise [47-49] and antiobesity drugs [50,51]. However, gene expression profiling of subcutaneous adipose tissue (SAT), visceral adipose tissue (VAT), BAT, and other tissues, including the liver and skeletal muscle [52], in the same individual after significant weight loss will allow us to delineate biological processes most likely related to weight loss. Moreover, it is observed that significant weight loss is associated with significant changes in blood pressure, TGs, HDL-C, and adiponectin. The multiple significant changes in glucose and lipid metabolism, as well as adipose tissue function in response to weight loss, are significant confounding factors, which may confound the observed gene expression changes, either in a concerted mode or as single factors [53]. To follow up the lipolysis and metabolic statues in VAT, SAT, BAT, and hepatocyte, biochemical variables related to metabolism will be measured in blood samples. In addition, to control for the energy expenditure, we will homogenize food intake for all groups. However, it is necessary that scientists should introduce beneficial interventions with high effectiveness and low side effects. To our knowledge, adenosine is present in adipose tissue after breakdown by ectoenzymes of ATP released as a cotransmitter from sympathetic nerves and adipocytes [19]. Adenosine has been shown to regulate hamster BAT respiration at an early metabolic step of the stimulus- thermogenesis sequence [54]. Adenosine increased lipolysis and induced thermogenesis in brown adipocytes via adenosine A2A receptors, and A2A agonists were shown to counteract high-fat diet–induced obesity in mice [55]. Thus, in this study, adenosine as an exogenous intervention will be used in terms of antiobesity-induced high-fat diet, and also synergistic impact of adenosine will be evaluated with combination of the type of exercise following high-fat diet. Moreover, lower serum 25-hydroxyvitamin D concentrations have been consistently linked to increasing BMI [56]. Prior studies also demonstrated that the loss of adiposity is associated with a proportional increase in circulating vitamin D levels [57]. Moreover, the stimulation of whole-body fat oxidation and the increase in fecal energy loss are 2 established mechanisms by which vitamin D is changing energy balance and may affect weight loss [58]. In this regard, another aspect of this study is the investigation of vitamin D3 injection as an inhibitor of obesity-induced high-fat diet. In addition, vitamin D3 injection will be evaluated along with the combination of type of exercise related to the exercise volume along with food and tap water ad libitum for the first 6 weeks of the exercise training phase, and after that, food will be homogenized in the next 6 weeks of the exercise training phase.

### Expected Results

The researcher expects to observe a decrease in anthropometric indices, such as weight of the rats, as apparent changes due to the isocaloric exercise training, drug injection, and food intake following the received high-fat diet. In addition, exercise related to energy consumption, vitamin D3, and adenosine, separately or interacting 2 interventions, may have effectiveness on molecular and biochemical changes in each metabolic tissue, which will result in weight loss. In addition, it is likely that biochemical and molecular changes and upregulation will be observed in line with the increase in lipolysis and beta oxidation in muscle and fat tissue as a result of performing isocaloric training in drug-receiving rats and groups on a high-fat diet.

## Abbreviations

ATP: adenosine triphosphate
BAT: brown adipose tissue
BMI: body mass index
cDNA: complementary DNA
GAPDH: glyceraldehyde-3-phosphate dehydrogenase
HDL-C: high-density lipoprotein cholesterol
HIIT: high-intensity interval training
IP: intraperitoneal
MIT: moderate-interval training
PCR: polymerase chain reaction
QUICKI: quantitative insulin sensitivity check index
SAT: subcutaneous adipose tissue
TG: total triglyceride
VAT: visceral adipose tissue

## References

[1] Mokdad AH, Ford ES, Bowman BA, Dietz WH, Vinicor F, Bales VS, Marks JS (2003). Prevalence of obesity, diabetes, and obesity-related health risk factors, 2001. J Am Med Assoc.

[2] James PT, Rigby N, Leach R, International Obesity Task Force (2004). The obesity epidemic, metabolic syndrome and future prevention strategies. Eur J Cardiovasc Prev Rehabil.

[3] Han TS, Lean ME (2016). A clinical perspective of obesity, metabolic syndrome and cardiovascular disease. JRSM Cardiovasc Dis.

[4] Huang L, Chen J, Cao P, Pan H, Ding C, Xiao T, Zhang P, Guo J, Su Z (2015). Anti-obese effect of glucosamine and chitosan oligosaccharide in high-fat diet-induced obese rats. Mar Drugs.

[5] Olshansky SJ, Passaro DJ, Hershow RC, Layden J, Carnes BA, Brody J, Hayflick L, Butler RN, Allison DB, Ludwig DS (2005). A potential decline in life expectancy in the United States in the 21st century. N Engl J Med.

[6] Li P, Oh DY, Bandyopadhyay G, Lagakos WS, Talukdar S, Osborn O, Johnson A, Chung H, Maris M, Ofrecio JM, Taguchi S, Lu M, Olefsky JM (2015). LTB4 promotes insulin resistance in obese mice by acting on macrophages, hepatocytes and myocytes. Nat Med.

[7] Lu B, Bridges D, Yang Y, Fisher K, Cheng A, Chang L, Meng Z, Lin JD, Downes M, Yu RT, Liddle C, Evans RM, Saltiel AR (2014). Metabolic crosstalk: molecular links between glycogen and lipid metabolism in obesity. Diabetes.

[8] Shimizu N, Maruyama T, Yoshikawa N, Matsumiya R, Ma Y, Ito N, Tasaka Y, Kuribara-Souta A, Miyata K, Oike Y, Berger S, Schütz G, Takeda S, Tanaka H (2015). A muscle-liver-fat signalling axis is essential for central control of adaptive adipose remodelling. Nat Commun.

[9] Bilski J, Mazur-Bialy A, Brzozowski B, Magierowski M, Jasnos K, Krzysiek-Maczka G, Urbanczyk K, Ptak-Belowska A, Zwolinska-Wcislo M, Mach T, Brzozowski T (2015). Moderate exercise training attenuates the severity of experimental rodent colitis: the importance of crosstalk between adipose tissue and skeletal muscles. Mediators Inflamm.

[10] Keenan MJ, Zhou J, Hegsted M, Pelkman C, Durham HA, Coulon DB, Martin RJ (2015). Role of resistant starch in improving gut health, adiposity, and insulin resistance. Adv Nutr.

[11] Si X, Strappe P, Blanchard C, Zhou Z (2017). Enhanced anti-obesity effects of complex of resistant starch and chitosan in high fat diet fed rats. Carbohydr Polym.

[12] Asher WL (1972). Mortality rate in patients receiving “diet pills”. Curr Ther Res Clin Exp.

[13] Fink KB, Göthert M (2007). 5-HT receptor regulation of neurotransmitter release. Pharmacol Rev.

[14] Antel J, Hebebrand J (2012). Weight-reducing side effects of the antiepileptic agents topiramate and zonisamide. Handb Exp Pharmacol.

[15] White MA, Grilo CM (2013). Bupropion for overweight women with binge-eating disorder: a randomized, double-blind, placebo-controlled trial. J Clin Psychiatry.

[16] Pooyandjoo M, Nouhi M, Shab-Bidar S, Djafarian K, Olyaeemanesh A (2016). The effect of (L-)carnitine on weight loss in adults: a systematic review and meta-analysis of randomized controlled trials. Obes Rev.

[17] Marcotorchino J, Tourniaire F, Astier J, Karkeni E, Canault M, Amiot M, Bendahan D, Bernard M, Martin J, Giannesini B, Landrier J (2014). Vitamin D protects against diet-induced obesity by enhancing fatty acid oxidation. J Nutr Biochem.

[18] Abbas MA (2017). Physiological functions of Vitamin D in adipose tissue. J Steroid Biochem Mol Biol.

[19] Chang E, Kim Y (2016). Vitamin D decreases adipocyte lipid storage and increases NAD-SIRT1 pathway in 3T3-L1 adipocytes. Nutrition.

[20] Lee H, Bae S, Yoon Y (2012). Anti-adipogenic effects of 1,25-dihydroxyvitamin D3 are mediated by the maintenance of the wingless-type MMTV integration site/β-catenin pathway. Int J Mol Med.

[21] Sakuma S, Fujisawa J, Sumida M, Tanigawa M, Inoda R, Sujihera T, Kohda T, Fujimoto Y (2012). The involvement of mitogen-activated protein kinases in the 1α,25-dihydroxy-cholecalciferol-induced inhibition of adipocyte differentiation in vitro. J Nutr Sci Vitaminol (Tokyo).

[22] Vu D, Ong JM, Clemens TL, Kern PA (1996). 1,25-Dihydroxyvitamin D induces lipoprotein lipase expression in 3T3-L1 cells in association with adipocyte differentiation. Endocrinology.

[23] Borissova AM, Tankova T, Kirilov G, Dakovska L, Kovacheva R (2003). The effect of vitamin D3 on insulin secretion and peripheral insulin sensitivity in type 2 diabetic patients. Int J Clin Pract.

[24] Narvaez CJ, Simmons KM, Brunton J, Salinero A, Chittur SV, Welsh JE (2013). Induction of STEAP4 correlates with 1,25-dihydroxyvitamin D3 stimulation of adipogenesis in mesenchymal progenitor cells derived from human adipose tissue. J Cell Physiol.

[25] Nimitphong H, Holick MF, Fried SK, Lee M (2012). 25-hydroxyvitamin D₃ and 1,25-dihydroxyvitamin D₃ promote the differentiation of human subcutaneous preadipocytes. PLoS One.

[26] Zabielska MA, Borkowski T, Slominska EM, Smolenski RT (2015). Inhibition of AMP deaminase as therapeutic target in cardiovascular pathology. Pharmacol Rep.

[27] Fredholm BB, IJzerman AP, Jacobson KA, Linden J, Müller CE (2011). International Union of Basic and Clinical Pharmacology. LXXXI. Nomenclature and classification of adenosine receptors--an update. Pharmacol Rev.

[28] Eisenstein A, Carroll SH, Johnston-Cox H, Farb M, Gokce N, Ravid K (2014). An adenosine receptor-Krüppel-like factor 4 protein axis inhibits adipogenesis. J Biol Chem.

[29] Johnston-Cox H, Eisenstein AS, Koupenova M, Carroll S, Ravid K (2014). The macrophage A2B adenosine receptor regulates tissue insulin sensitivity. PLoS One.

[30] Johnston-Cox H, Koupenova M, Yang D, Corkey B, Gokce N, Farb MG, LeBrasseur N, Ravid K (2012). The A2b adenosine receptor modulates glucose homeostasis and obesity. PLoS One.

[31] Barankiewicz J, Cohen A (1985). Purine nucleotide metabolism in resident and activated rat macrophages in vitro. Eur J Immunol.

[32] Chambliss HO (2005). Exercise duration and intensity in a weight-loss program. Clin J Sport Med.

[33] Perry CG, Heigenhauser GJ, Bonen A, Spriet LL (2008). High-intensity aerobic interval training increases fat and carbohydrate metabolic capacities in human skeletal muscle. Appl Physiol Nutr Metab.

[34] da Rocha GL, Crisp AH, de Oliveira MR, da Silva CA, Silva JO, Duarte AC, Sene-Fiorese M, Verlengia R (2016). Effect of high intensity interval and continuous swimming training on body mass adiposity level and serum parameters in high-fat diet fed rats. ScientificWorldJournal.

[35] Trapp EG, Chisholm DJ, Freund J, Boutcher SH (2008). The effects of high-intensity intermittent exercise training on fat loss and fasting insulin levels of young women. Int J Obes (Lond).

[36] Martins C, Kazakova I, Ludviksen M, Mehus I, Wisloff U, Kulseng B, Morgan L, King N (2016). High-intensity interval training and isocaloric moderate-intensity continuous training result in similar improvements in body composition and fitness in obese individuals. Int J Sport Nutr Exerc Metab.

[37] Vina J, Sanchis-Gomar F, Martinez-Bello V, Gomez-Cabrera MC (2012). Exercise acts as a drug; the pharmacological benefits of exercise. Br J Pharmacol.

[38] Pereira MG, Ferreira JC, Bueno CR, Mattos KC, Rosa KT, Irigoyen MC, Oliveira EM, Krieger JE, Brum PC (2009). Exercise training reduces cardiac angiotensin II levels and prevents cardiac dysfunction in a genetic model of sympathetic hyperactivity-induced heart failure in mice. Eur J Appl Physiol.

[39] Ferreira J, Rolim N, Bartholomeu J, Gobatto C, Kokubun E, Brum P (2007). Maximal lactate steady state in running miceffect of exercise training. Clin Exp Pharmacol Physiol.

[40] Muniyappa R, Chen H, Muzumdar RH, Einstein FH, Yan X, Yue LQ, Barzilai N, Quon MJ (2009). Comparison between surrogate indexes of insulin sensitivity/resistance and hyperinsulinemic euglycemic clamp estimates in rats. Am J Physiol Endocrinol Metab.

[41] Vilchis-Gil J, Klünder-Klünder M, Flores-Huerta S (2018). Effect on the metabolic biomarkers in schoolchildren after a comprehensive intervention using electronic media and in-person sessions to change lifestyles: community trial. J Med Internet Res.

[42] Bernardis LL (1970). Prediction of carcass fat, water and lean body mass from Lee's “nutritive ratio” in rats with hypothalamic obesity. Experientia.

[43] Phillis JW (2004). Adenosine and adenine nucleotides as regulators of cerebral blood flow: roles of acidosis, cell swelling, and KATP channels. Crit Rev Neurobiol.

[44] Jackson EK (2004). Intraperitoneal administration of adenosine inhibits formation of abdominal adhesions. Dis Colon Rectum.

[45] Bish CL, Blanck HM, Serdula MK, Marcus M, Kohl HW, Khan LK (2005). Diet and physical activity behaviors among Americans trying to lose weight: 2000 Behavioral Risk Factor Surveillance System. Obes Res.

[46] Hill C, Weir BW, Fuentes LW, Garcia-Alvarez A, Anouti DP, Cheskin LJ (2018). Relationship between weekly patterns of caloric intake and reported weight loss outcomes: retrospective cohort study. JMIR Mhealth Uhealth.

[47] Meckling KA, O'Sullivan C, Saari D (2004). Comparison of a low-fat diet to a low-carbohydrate diet on weight loss, body composition, and risk factors for diabetes and cardiovascular disease in free-living, overweight men and women. J Clin Endocrinol Metab.

[48] Donnelly JE, Hill JO, Jacobsen DJ, Potteiger J, Sullivan DK, Johnson SL, Heelan K, Hise M, Fennessey PV, Sonko B, Sharp T, Jakicic JM, Blair SN, Tran ZV, Mayo M, Gibson C, Washburn RA (2003). Effects of a 16-month randomized controlled exercise trial on body weight and composition in young, overweight men and women: the Midwest Exercise Trial. Arch Intern Med.

[49] Foster-Schubert KE, Alfano CM, Duggan CR, Xiao L, Campbell KL, Kong A, Bain CE, Wang C, Blackburn GL, McTiernan A (2012). Effect of diet and exercise, alone or combined, on weight and body composition in overweight-to-obese postmenopausal women. Obesity (Silver Spring).

[50] Kim GW, Lin JE, Blomain ES, Waldman SA (2014). Antiobesity pharmacotherapy: new drugs and emerging targets. Clin Pharmacol Ther.

[51] Onakpoya IJ, Heneghan CJ, Aronson JK (2016). Post-marketing withdrawal of anti-obesity medicinal products because of adverse drug reactions: a systematic review. BMC Med.

[52] Furukawa S, Fujita T, Shimabukuro M, Iwaki M, Yamada Y, Nakajima Y, Nakayama O, Makishima M, Matsuda M, Shimomura I (2004). Increased oxidative stress in obesity and its impact on metabolic syndrome. J Clin Invest.

[53] Mardinoglu A, Heiker JT, Gärtner D, Björnson E, Schön MR, Flehmig G, Klöting N, Krohn K, Fasshauer M, Stumvoll M, Nielsen J, Blüher M (2015). Extensive weight loss reveals distinct gene expression changes in human subcutaneous and visceral adipose tissue. Sci Rep.

[54] Schimmel RJ, McCarthy L (1984). Role of adenosine as an endogenous regulator of respiration in hamster brown adipocytes. Am J Physiol.

[55] Gnad T, Scheibler S, von Kügelgen I, Scheele C, Kilić A, Glöde A, Hoffmann LS, Reverte-Salisa L, Horn P, Mutlu S, El-Tayeb A, Kranz M, Deuther-Conrad W, Brust P, Lidell ME, Betz MJ, Enerbäck S, Schrader J, Yegutkin GG, Müller CE, Pfeifer A (2014). Adenosine activates brown adipose tissue and recruits beige adipocytes via A2A receptors. Nature.

[56] Pereira-Santos M, Costa PR, Assis AM, Santos CA, Santos DB (2015). Obesity and vitamin D deficiency: a systematic review and meta-analysis. Obes Rev.

[57] Pannu PK, Zhao Y, Soares MJ (2016). Reductions in body weight and percent fat mass increase the vitamin D status of obese subjects: a systematic review and metaregression analysis. Nutr Res.

[58] Gonzalez JT, Rumbold PL, Stevenson EJ (2012). Effect of calcium intake on fat oxidation in adults: a meta-analysis of randomized, controlled trials. Obes Rev.

